# Generative AI and cognitive load in education: a systematic review of WoS/SSCI-indexed studies through the lens of cognitive load theory

**DOI:** 10.3389/fpsyg.2026.1921504

**Published:** 2026-08-27

**Authors:** Weixu Qian, Feng Yang, Yaming Cao, Liangliang Yi, Run Gu, Zhuo Wang

**Affiliations:** 1 School of Education, Ludong University, Yantai, China; 2 School of Art & Design, Guangzhou Polytechnic University, Guangzhou, China; 3 School of Literature and Journalism & Communication, Hanshan Normal University, Chaozhou, China; 4 College of Educational Sciences, Qingdao University, Qingdao, China

**Keywords:** cognitive load theory, cognitive offloading, extraneous load, generative artificial intelligence, germane load, higher education

## Abstract

Generative artificial intelligence (GenAI), together with closely related educational AI systems retrieved by the executed search, has been rapidly adopted across education, prompting empirical work on its consequences for learners' cognitive load. This systematic review of English-language, SSCI-indexed journal articles in the Web of Science Core Collection examined 39 empirical studies through the tripartite architecture of Cognitive Load Theory (CLT): intrinsic, extraneous, and germane load. Studies were dual-coded across 14 dimensions. The corpus was strongly experimental (28 of 39 studies) and dominated by undergraduate samples. Sixteen studies measured extraneous load as a distinct subtype: seven reported lower extraneous load, three reported no difference, one reported higher extraneous load, and one reported a curvilinear pattern under the reported AI/GenAI contrast; four did not provide a directional comparison. A separate 22 studies reported an overall or undifferentiated index, for which 11 reported lower load, five reported no difference, and two reported higher load, with four non-directional cases. These groups must not be pooled as evidence that AI or GenAI specifically reduces extraneous load. Eleven studies included a germane-load subscale, but only five yielded a directional comparison (four higher and one curvilinear). The modal overall verdict was conditional rather than uniformly beneficial (21 conditional, 16 beneficial, two mixed): benefits depended on scaffolding, dosage, learner prior knowledge, and task design. Only 13 studies framed AI/GenAI through cognitive offloading. We therefore argue that future research should measure the CLT components separately, report the AI/GenAI contrast transparently, and use an offloading lens to distinguish support for learning from substitution for learning.

## Introduction

The diffusion of generative artificial intelligence (GenAI) into classrooms has been faster than that of perhaps any prior educational technology. Within 2 years of the public release of large language models, empirical studies appeared across the full span of education—from K-12 science to graduate writing instruction, from programming to art appreciation—each asking some version of the same question: what does GenAI do to the mind of the learner? Hu et al. (2025) situated this question in college art appreciation; researchers working in higher education have linked GenAI feedback to self-efficacy and knowledge construction (Tu et al., 2026; Xu X. et al., 2025); and others have traced its consequences for higher-order thinking and academic enjoyment (Zhang and Jiang, 2026). Beneath this thematic diversity lies a shared theoretical anxiety, namely that the cognitive consequences of GenAI are double-edged.

That anxiety is well founded. On one reading, GenAI is the most powerful instructional-design technology yet devised for managing the limits of working memory: it can decompose complex tasks, supply worked examples on demand, and translate dense material into accessible explanations, all of which Cognitive Load Theory (CLT) predicts should lower the extraneous demands that impede learning (Sun and Liu, 2026b; Qi et al., 2026). On a competing reading, the very fluency of GenAI invites learners to outsource the effortful cognition that learning requires—to let the machine think—so that tasks feel easier precisely because less learning is taking place (Liu D. et al., 2026; Wang and Hew, 2026). These two readings are not merely optimistic and pessimistic moods; they correspond to genuinely different cognitive mechanisms, and they can be distinguished empirically. The difficulty is that the empirical literature has grown faster than its own capacity for synthesis.

Three problems impede a cumulative account. First, cognitive load is operationalized inconsistently: some studies report the full intrinsic/extraneous/germane triad, others a single overall index, and still others treat load only as a theoretical motivation without measuring it. Second, the studies differ in the causal role they assign to load—outcome, mediator, moderator, or antecedent—so that ostensibly comparable findings are not in fact commensurable. Third, the field is split between an instructional-design tradition, which treats GenAI as a tool for load management, and an emerging cognitive-offloading tradition, which treats GenAI as a substitute for cognition; these traditions rarely cite one another. The result is a literature that is individually rigorous but collectively inconclusive.

This review addresses that fragmentation by imposing a single theoretical spine—the tripartite structure of CLT—on the empirical corpus, and by coding every study against a common, decision-rule-based framework so that findings can be compared on equal terms. We synthesize 39 empirical studies of GenAI and closely related educational AI systems retrieved by the executed search, asking not only whether the reported AI/GenAI contrast raises or lowers load, but which load, measured how, under what conditions, and with what theoretical framing. In doing so we aim to convert a scattered body of findings into a structured account of what is known, what is contested, and what remains unexamined.

The contribution of this review is therefore as much diagnostic as descriptive. Prior narrative treatments have tended to ask whether GenAI “helps” learning, a framing that invites the very conflation this review seeks to dissolve. By insisting on the componential structure of load, and by reading each study's load result in light of how that load was measured and what causal role it played, we can specify not only the balance of evidence but the conditions under which the evidence can be trusted to mean what it appears to mean. Where a study reports an undifferentiated load reduction, we treat the finding as real but interpretively incomplete; where a study decomposes the triad, we can say something about learning and not merely about ease. To assemble a defensible evidence base, we drew on the Web of Science (WoS) Core Collection, the database our group has used for prior systematic reviews in educational technology (Guan et al., 2022; Sun et al., 2023; Tang et al., 2026); WoS is also widely used as a source for bibliometric and review research (Birkle et al., 2020; Li et al., 2018), making it a defensible source for this deliberately bounded corpus.

The review is guided by three research questions concerning measurement (RQ1), the direction and consistency of GenAI's effects on the three load components (RQ2), and the field's engagement with the cognitive-offloading perspective (RQ3). By foregrounding germane load and the offloading mechanism—two facets the literature has most neglected—we seek to identify the specific evidentiary gaps that the next generation of studies must close.

This review is guided by three research questions:

RQ1: How have empirical studies operationalized and measured cognitive load when investigating GenAI and related educational AI systems in educational settings?
RQ2: What is the direction of the reported AI/GenAI contrast on the three components of cognitive load (intrinsic, extraneous, germane), and how consistent is the evidence?
RQ3: To what extent does the literature engage the cognitive-offloading perspective, and what theoretical and methodological gaps remain?

## Theoretical framework

Cognitive Load Theory provides the organizing framework for this review. Originating in Sweller's (1988) analysis of problem solving and schema acquisition, and developed in subsequent architectural accounts (Sweller et al., 1998, 2019), CLT begins from the premise that working memory is severely limited in both capacity and duration, whereas long-term memory, organized as schemas, is effectively unlimited. Learning consists in constructing and automating schemas, and instruction succeeds or fails according to how it manages the load imposed on working memory during this process. The theory's enduring contribution is its decomposition of that load into functionally distinct components.

Intrinsic load arises from the inherent complexity of the material relative to the learner's prior knowledge—from the number of interacting elements that must be held in mind simultaneously. Extraneous load is imposed by the manner of presentation and by task features irrelevant to learning; it is the load that good instructional design seeks to minimize. Germane load, in the formulation most studies in this corpus adopt, refers to the working-memory resources devoted to the effortful processing that actually builds schemas—the productive load that learning requires. The pedagogical ideal is therefore not to minimize load wholesale but to minimize extraneous load so that capacity is freed for germane processing, while keeping intrinsic load within manageable bounds through sequencing and scaffolding.

This tripartite structure maps naturally onto the central tension surrounding GenAI. If GenAI functions as an instructional-design aid—structuring tasks, supplying examples, clarifying complex content—its primary effect should be to reduce extraneous load and, ideally, to redirect the freed capacity toward germane processing. The studies in this corpus that report reduced extraneous load alongside maintained or improved learning are consistent with this account (Sun and Liu, 2026a,b; Qi et al., 2026). If, however, GenAI functions as a cognitive substitute, learners may experience lower load not because extraneous demands have been stripped away but because the germane processing itself has been offloaded to the machine. The two cases are observationally similar at the level of an overall load score yet opposite in their implications for learning, and they can be separated only by measuring germane load directly.

For this reason we extend the CLT spine with the cognitive-offloading perspective (Risko and Gilbert, 2016), which conceptualizes the use of external tools to reduce internal cognitive demand and draws attention to its costs as well as its benefits. The offloading account is not a rival to CLT but a complement to it: CLT specifies the structure of the load that is reduced, while offloading specifies the mechanism and the locus of that reduction—whether the cognition has been supported from outside or relocated outside. The distinction is consequential for GenAI specifically, because a conversational system that can produce a finished argument, a working program, or a polished paragraph offers learners an unprecedented opportunity to relocate, rather than merely lighten, the cognitive work of a task. Grounded-theory work in this corpus on AI-assisted programming captured precisely this dynamic, distinguishing AI as tool, tutor, and crutch and observing that offloading becomes visible when “both extraneous and germane processing are minimized” (Liu D. et al., 2026). Reading the corpus through both lenses—CLT for the structure of load and offloading for the mechanism of its reduction—allows this review to ask not merely whether GenAI lowers load, but whether the load it lowers is the kind that should be lowered.

## Method

This review followed a systematic procedure encompassing corpus assembly, screening, framework development, dual coding, and synthesis. The corpus was assembled from a defined set of empirical papers on GenAI and related educational AI systems captured by the executed Topic-field search, supplemented by backward snowballing from the reference lists of the most central studies. Snowballed candidates were retained only after full-text screening against the inclusion criteria, rather than on the basis of title and abstract alone.

Search strategy. The literature search was conducted in the Web of Science (WoS) Core Collection. The review was deliberately restricted to peer-reviewed, English-language articles indexed in the Social Sciences Citation Index (SSCI) and filtered to the WoS Education and Educational Research category. It is therefore a systematic review of this defined WoS/SSCI corpus, not a claim of exhaustive coverage of all AI-and-cognitive-load research. The search was entered in three Topic fields, joined with AND: Topic 1: “cognitive load” or “mental effort” or “mental load” or “cognitive burden;” Topic 2: “Artificial intelligence” or AI or “generative AI” or “large language models” or chatgpt or GPT or chatbot or “conversational AI;” Topic 3: university or college or undergraduate or graduate or pre-service or freshmen or sophomore or “higher education.” No Author-field criterion was used. The final search was conducted in June 2026, with no publication-year restriction (database inception to the search month). The recorded filters were English, Article, SSCI, and Education and Educational Research. A saved WoS query link is supplied in the Supplementary material as an access aid for readers with appropriate database access; it is not the sole audit record. Backward citation screening of central included studies was used as a supplementary check. The review was not preregistered. The full strategy, filters, selection records, and PRISMA 2020 checklist (Page et al., 2021) are supplied in the Supplementary material.

**Figure 1:**
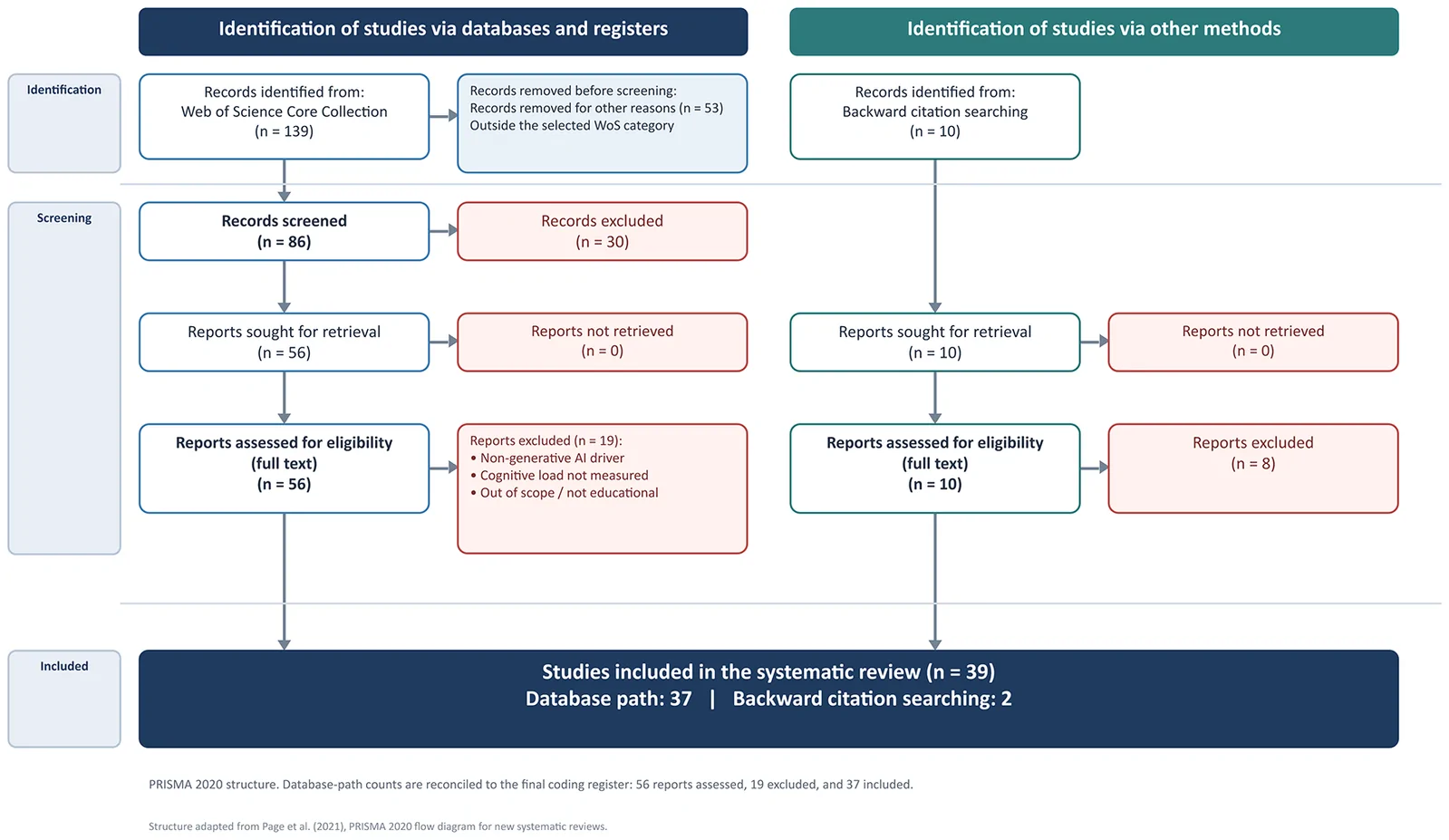
PRISMA 2020 flow diagram of the study selection process, from identification through screening and eligibility to the final included corpus (*N* = 39). Adapted from Page et al. (2021), licensed under CC BY 4.0. Source: https://www.prisma-statement.org/prisma-2020-flow-diagram.

The database search yielded 139 records. After 53 records outside the selected WoS category were removed, 86 records remained for screening. Thirty records were excluded during title-and-abstract screening, and 56 reports were sought, retrieved, and assessed in full text. Nineteen full-text reports were excluded with item-level reasons, leaving 37 studies from the database-search pathway. Backward citation screening contributed two additional included studies, yielding the final corpus of 39 studies. Figure 1 reports the same selection counts.

At the screening and eligibility stages, reports were excluded principally because the AI system lay outside the review's executed AI/GenAI scope (for example, a BERT classifier, retrieval-based chatbot, or curated knowledge-base system), cognitive load was invoked only as a theoretical motivation rather than measured or coded, or the report lay outside the educational scope. Supplementary Table S1 identifies each of the 39 included studies by full citation and a verified DOI link. Supplementary Table S2 lists all 19 database-path full-text exclusions in the final coding register and their standardized reasons. The included studies not individually cited elsewhere in the main text are listed in full in Supplementary Table S1 (Chen and Chang, 2024; Zhang et al., 2024; Huang et al., 2025; Li et al., 2025; Liu et al., 2025; Nguyen et al., 2025; Wu and Ho, 2025; Jou and Hao, 2026; Lu and Ba, 2026; Lukešová and Jennings, 2026; Xiong, 2026; Xu et al., 2026; Zheng et al., 2026).

Inclusion required that a study (a) examine a generative AI system or a closely related educational AI/conversational system falling within the executed Topic-field query, as the intervention or independent variable; (b) measure or thematically code cognitive load, mental effort, or cognitive offloading, rather than invoke load only as a theoretical motivation; and (c) report primary empirical data, whether quantitative or qualitative, in an educational or learning context. Applying these criteria removed a substantial number of initially plausible papers—several in which the AI driver proved outside the review's defined scope, and several in which cognitive load was named but never measured—underscoring the importance of full-text re-screening. The final corpus comprised 39 studies.

A coding framework of 14 dimensions was developed with the tripartite CLT structure as its spine, covering identification and scope, study descriptors, design and method, the measurement and reported components of load, the causal role of load in each study's model, the direction of GenAI's effect on extraneous and germane load, the presence of cognitive-offloading framing, the co-measured learning outcome, and each study's net verdict. Because earlier coding attempts had foundered on ambiguous category boundaries, every dimension was specified as a forced-choice decision tree with explicit tiebreakers, prohibiting intermediate or mixed codes wherever a determinate rule could be written.

Two raters coded all studies independently. Inter-coder reliability, computed as Cohen's kappa across the categorical dimensions, was substantial to almost perfect. Three dimensions that initially fell below the 0.80 threshold—the measurement instrument, the offloading framing, and the net verdict—had their decision rules revised rather than being adjudicated case by case, after which they were re-coded; the offloading dimension, recast as a closed-list keyword test, reached perfect agreement. All disagreements were resolved to consensus, and a disagreement log was retained for transparency.

No validated study-level risk-of-bias or methodological-quality appraisal was preserved in the original review record. To avoid retrospectively presenting a descriptive coding profile as if it were a formal MMAT, JBI, or risk-of-bias assessment, we do not score or quality-weight the included studies in this revision. Supplementary Table S3 instead provides a study-level design-and-measurement profile to make the basis for interpretation transparent. The absence of a formal, independent appraisal is retained as a limitation of the review.

Synthesis used a structured descriptive evidence map within the CLT framework. For each measurement group, we report the direction stated in the primary study, distinguish directional contrasts from non-directional or qualitative cases, and keep extraneous-specific, overall/undifferentiated, and germane findings separate. These counts are not effect-size pooling, not a statistical-significance vote count, and not precision- or quality-weighted estimates. Heterogeneity of instruments, interventions, comparison conditions, and effect metrics precluded a defensible meta-analysis.

## Results

### Measurement of cognitive load (RQ1)

The corpus revealed marked heterogeneity in how cognitive load was operationalized, which directly conditions the comparability of its findings. No single instrument dominated. The most frequent approaches were the mental-load-and-mental-effort scales in the tradition of Hwang and colleagues and a range of other validated scales, each used in nine studies, followed by single-item mental-effort measures in the tradition of Paas (1992) and Paas et al. (2003; seven studies). The full three-factor instruments that operationalize the CLT triad directly—chiefly the Leppink et al. (2013) and Klepsch et al. (2017) scales—appeared in only six studies combined (Li et al., 2026; Huang et al., 2026). Four studies relied on the NASA Task Load Index, three coded load as a qualitative theme, and one used author-constructed items.

**Figure 2:**
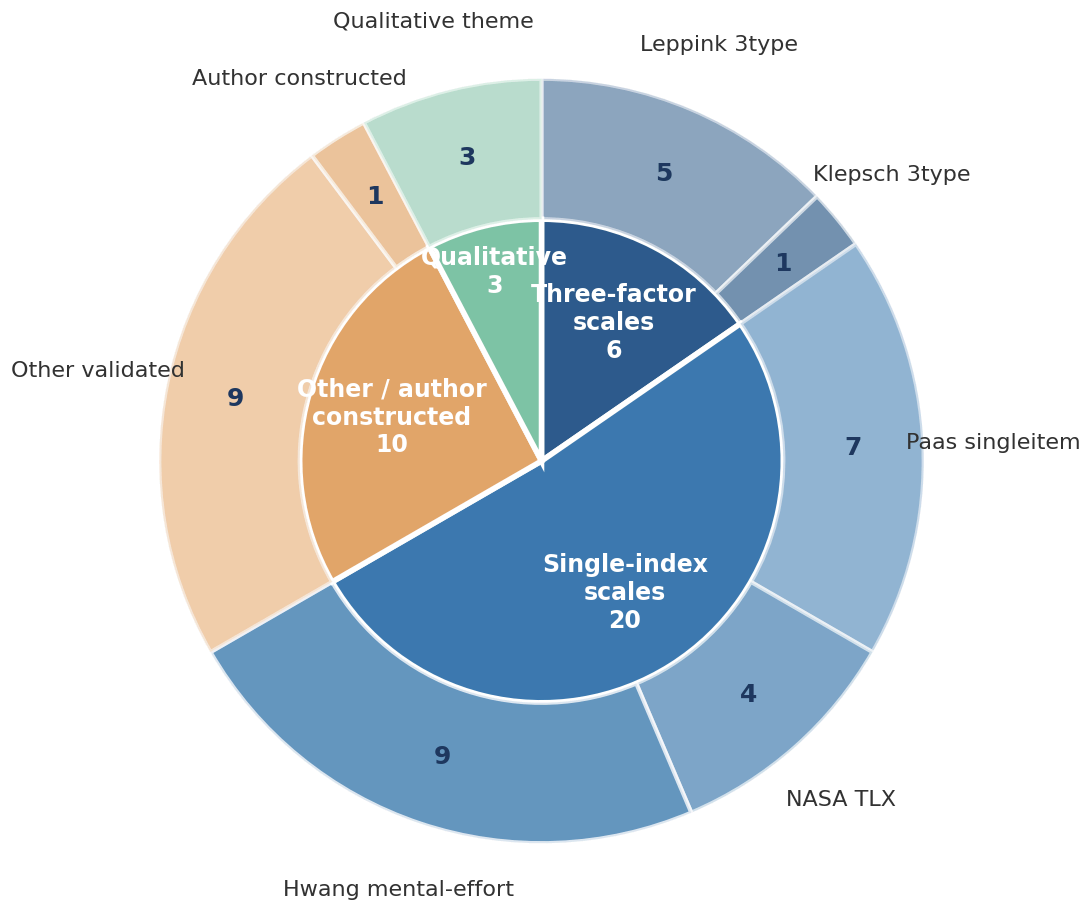
Distribution of cognitive load measurement instruments across the 39 included studies, grouped by measurement family.

This dispersion has a consequence that is easy to overlook: 22 of the 39 studies reported only an overall or undifferentiated load index rather than separate intrinsic, extraneous, and germane scores. When a study reports that an AI/GenAI contrast lowered “cognitive load” without decomposing that load, a decrease driven by stripped-away extraneous demands is indistinguishable from a decrease driven by offloaded germane processing. The measurement choices of the field therefore pre-empt the very question the field most needs to answer. As illustrated in Figure 2, instrument choice is fragmented, and the three-factor scales best suited to CLT remain a minority practice.

### Research designs and sample characteristics

The corpus was strongly experimental. Of the 39 studies, 28 employed experimental designs with a manipulated GenAI condition and a comparison group or counterbalanced contrast, seven were survey-correlational studies modeling load through structural equation or regression methods, two were single-group pre-post designs, and two were qualitative. This experimental weighting lends the corpus comparatively strong internal validity for causal claims about GenAI's effect on load, though it also means that the naturalistic, longitudinal dynamics of sustained GenAI use are under-represented.

**Figure 3:**
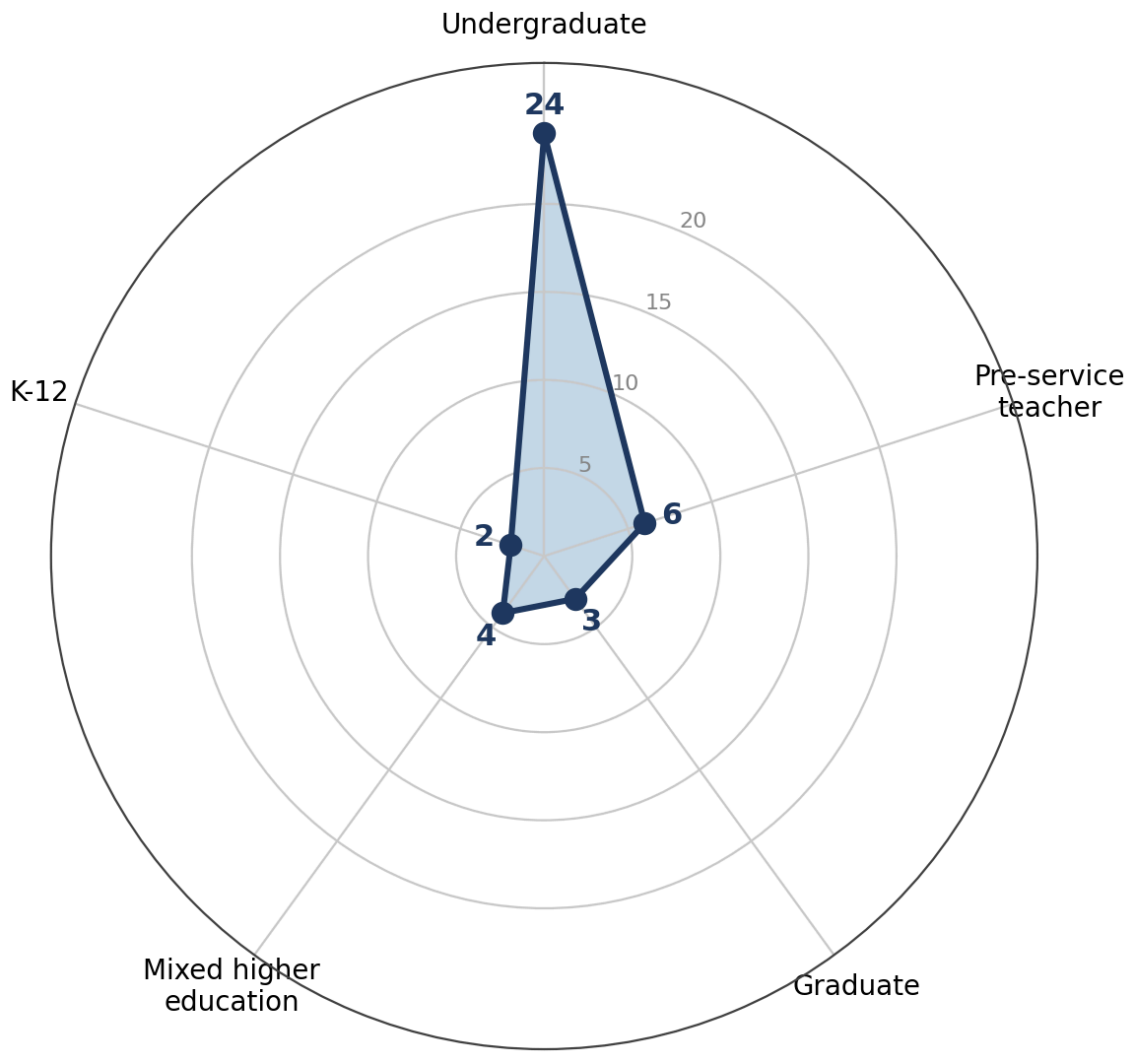
Educational levels represented across the 39 included studies.

Samples skewed toward higher education. Undergraduates were the modal population (24 studies), followed by pre-service teachers (six studies), with graduate, mixed-higher-education, and K-12 samples making up the remainder; only two studies examined school-age learners. Sample sizes ranged widely, from 23 to 951 participants (median 74), reflecting the mix of small controlled experiments (Li et al., 2024; Joo et al., 2026) and large survey studies (Zhang and Jiang, 2026; Liu P. et al., 2026). Subject domains clustered in language and writing (11 studies) and general or cross-domain instruction (9 studies), with smaller representations in social science and education, STEM, and the arts. As Figure 3 shows, this corpus speaks most directly to undergraduate learners and least to children, a distribution that should temper generalization to compulsory schooling.

### The direction of GenAI's effect on extraneous load (RQ2)

We report the direction of GenAI's reported effect separately by measurement group because combining them would erase the theoretical distinction this review is designed to test. Sixteen studies measured extraneous load as a distinct subtype: seven reported lower extraneous load, three no difference, one higher extraneous load, and one a curvilinear pattern; three supplied no directional GenAI comparison and one was qualitative. A separate 22 studies reported only an overall or undifferentiated index: 11 reported lower load, five no difference, two higher load, three no directional GenAI comparison, and one qualitative account. One further study measured intrinsic load only. Thus, across the 38 studies in the first two groups, the descriptive totals are 18 lower, eight showing no difference, three higher, one curvilinear, and eight non-directional or qualitative cases. These figures do not establish that GenAI reduces extraneous load specifically; only the first group bears on that component.

The strongest instances of this pattern come from controlled experiments that decomposed load into its components. Sun and Liu (2026b) found that an AI-supported group reported significantly lower extraneous load and, crucially, significantly higher germane load than a comparison group, a result echoed by (Sun and Liu 2026a), whose AI group achieved “a more optimal cognitive load balance (lower extraneous, higher germane load).” Using the Leppink three-factor scale, Guan et al. (2026) reported significantly lower intrinsic and extraneous load in chatbot-supported reading, and, under self-paced AIGC-supported questioning, Jing et al. (2025) found that AI-generated content “reduced extraneous cognitive load, increased germane cognitive load, and improved learning performance.” Studies using single overall indices converged on the same direction: Hu et al. (2025) reported significantly lower load in the experimental group in art appreciation, Hong and Guo (2025) observed a significant load reduction across experimental conditions in language learning, and Urban et al. (2024), in a controlled experiment, found that learners with ChatGPT assistance perceived the task as easier (*d* = 0.56) and requiring less mental effort (*d* = 0.58). The recurrent design feature in these load-relieving studies is structure: guiding questions, step-by-step prompts, worked examples, and decomposition of complex tasks, as in the AI-agent scaffolding described by Wang et al. (2025).

The three studies reporting increased load are instructive rather than anomalous. They tend to involve GenAI configurations that add coordination or evaluation costs. Zhang et al. (2026) found that an AI-feedback group reported significantly higher extraneous load than a teacher-feedback group, plausibly because learners had to interpret and reconcile machine feedback; Cheng et al. (2026) observed a significant pre-to-post rise in load under sustained AI use; and Wang and Hew (2026), while reporting greater writing performance, also found significantly greater mental effort under AI coaching—an increase the authors read as productive rather than wasteful. Where learners must monitor, verify, or integrate AI output, in other words, the management of the AI itself becomes a source of extraneous load. The single curvilinear association, in a large survey of AI dependency by Zhang and Jiang (2026), locates the lowest extraneous load at moderate rather than maximal reliance, implying that both under- and over-use of GenAI can raise the cost of task management.

**Figure 4:**
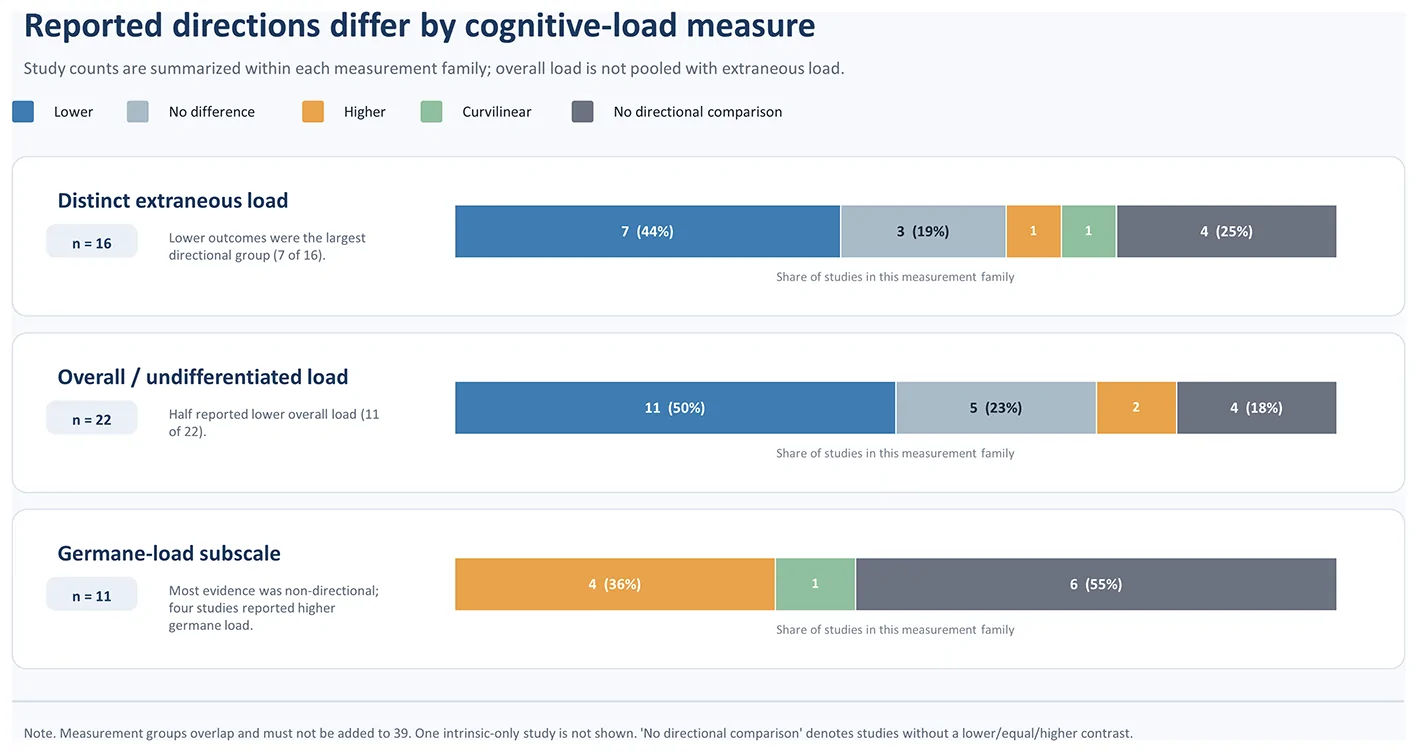
Direction reported for GenAI-related cognitive-load findings, shown separately for studies measuring distinct extraneous load (*n* = 16), overall/undifferentiated load (*n* = 22), and a germane-load subscale (*n* = 11). Panels overlap and cannot be summed to 39; one intrinsic-only study is not displayed.

Taken together, the evidence map supports a qualified interpretation rather than a pooled effect claim. Lower reported load is more common than higher reported load in both measurement groups, but the pedagogical meaning of an overall-load decrease remains indeterminate. The pattern should therefore be read alongside the instrument, comparison condition, and study design—not as quality-weighted evidence and not as a substitute for a formal risk-of-bias assessment. Figure 4 displays the separate denominators and makes clear that the panels overlap and cannot be added to 39.

### The neglect of germane load

Germane-load evidence is limited principally by reporting rather than by a complete absence of measurement. Eleven of the 39 studies employed a germane-load subscale. Of those 11, four reported higher germane load and one a curvilinear pattern under the relevant GenAI contrast; five did not provide a directional GenAI comparison and one was qualitative. Thus, only five studies yielded a directional comparative finding for germane load, while 28 studies did not measure a germane subscale at all. This remains a consequential gap because germane load indexes the schema-building processing that distinguishes learning from mere task completion.

The few studies that did measure germane load are correspondingly valuable. Sun and Liu (2026a,b) found germane load not merely preserved but significantly elevated in the AI condition even as extraneous load fell, the signature of an ideal load reallocation. Huang et al. (2026), using the Klepsch three-factor scale, reported that an AI chatbot ‘significantly stimulated the germane cognitive load' while reducing intrinsic load, and Jing et al. (2025) likewise paired reduced extraneous with increased germane load and improved performance under self-paced AIGC-supported questioning. The curvilinear exception is again Zhang and Jiang (2026), who found germane load—like higher-order thinking and academic enjoyment—peaking at moderate AI dependency and declining at the extremes, a pattern that an instructional-design account alone cannot easily explain.

The implication is sharp. A reported reduction in overall load remains ambiguous between two opposite possibilities: GenAI may free working-memory capacity for deeper processing, or it may absorb the deep processing itself. The five directional germane comparisons provide the only direct comparative evidence on whether germane processing changes under GenAI, and their small number precludes a general claim. For the field as a whole, the central pedagogical question—does GenAI make room for learning or stand in for it?—remains empirically open. This is a gap in measurement and reporting practice: validated multidimensional instruments exist, but only a minority of studies used them and still fewer reported a directional GenAI comparison.

### Engagement with cognitive offloading (RQ3)

The corpus's theoretical framing compounds the measurement gap. Only 13 of the 39 studies framed GenAI through cognitive offloading—using concepts such as offloading, outsourcing of thinking, the cognitive crutch, or metacognitive laziness—while 26 treated load purely as an instructional-design variable.

The studies that did adopt the offloading lens are, tellingly, among those most alert to GenAI's risks. The clearest case is the grounded-theory study by Liu D. et al. (2026), whose title—tool, tutor, or crutch—names the spectrum directly and whose analysis states that ‘offloading is evident when both extraneous and germane processing are minimized,' precisely the configuration in which a falling load score signals lost learning rather than efficient design. Ji et al. (2025) described a ChatGPT condition in which learners ‘used ChatGPT to offload cognitive resources in order to analyze and solve complex problems,' explicitly framing the load reduction as a transfer of work. Chen et al. (2026) reported the inverse surprise—an experimental group showing increased anxiety and cognitive load, possibly due to heightened metacognitive awareness—while Xu X. et al. (2025) found that metacognitive support did not move achievement yet altered learners' self-regulatory engagement, a reminder that the cognitive consequences of GenAI are not exhausted by a single load score. Even where the offloading framing accompanied a positive verdict, as in Bai (2026) and Wang and Hew (2026), it introduced the question of what the learner was no longer doing.

This distribution matters because the offloading perspective supplies exactly the interpretive apparatus the measurement gap leaves missing. Where instructional-design framing treats any load reduction as a success, the offloading lens insists on asking what cognition the reduction displaced. The relative rarity of this framing—present in only a third of the corpus—helps explain why so much of the literature reports load reductions without alarm: absent the conceptual vocabulary of offloading, a lower load score reads unambiguously as good news. Bringing the offloading lens into wider use, and pairing it with germane-load measurement, would allow the field to tell desirable from undesirable load reduction rather than conflating them.

### The causal role of cognitive load in study models

**Figure 5:**
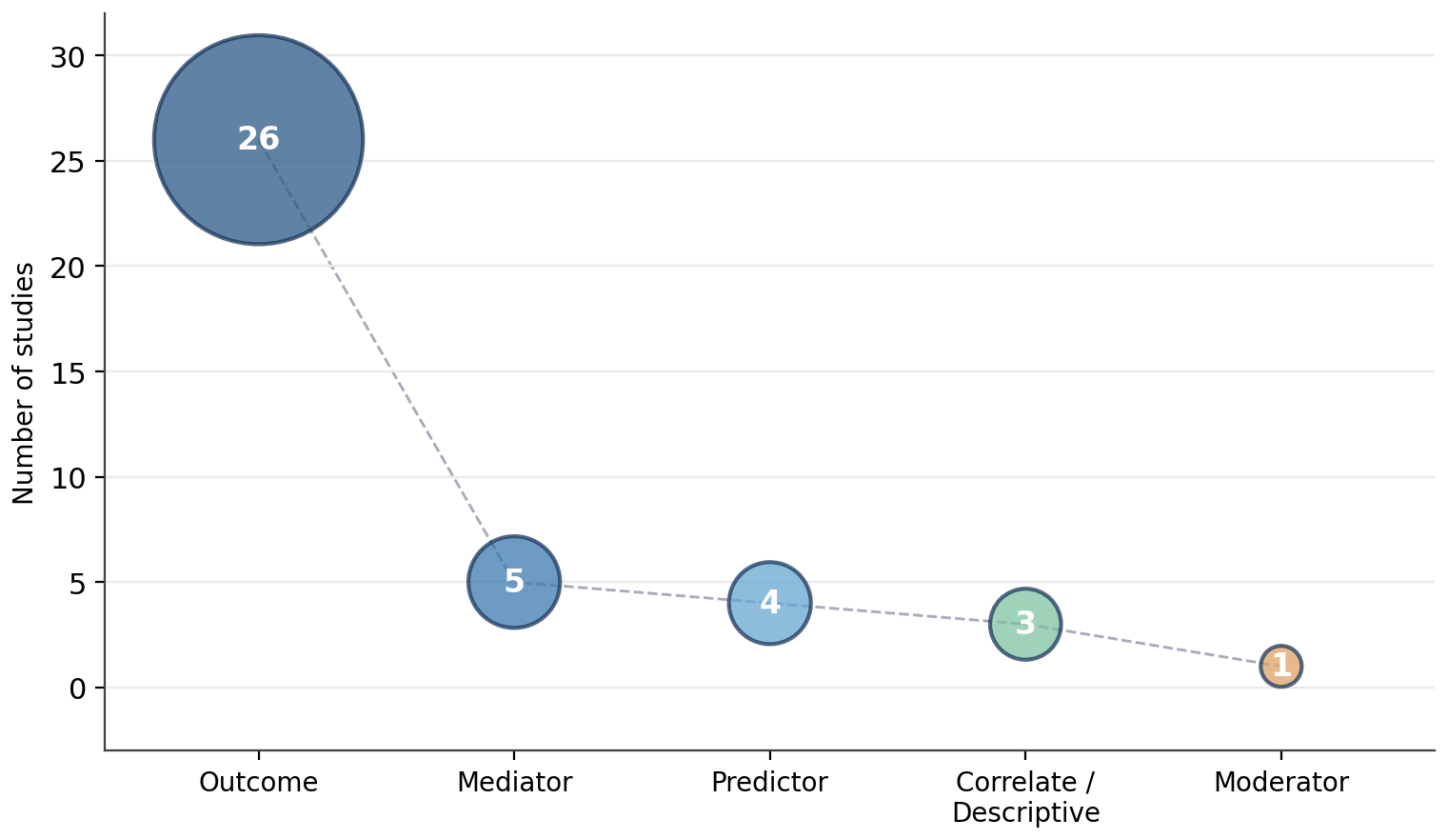
Causal role assigned to cognitive load across the 39 included studies.

A further source of incommensurability lies in the causal role each study assigned to cognitive load. As Figure 5 shows, in 26 of the 39 studies load functioned as an outcome—a dependent variable compared across GenAI and comparison conditions. In five it was positioned as a mediator or pathway variable, although not all of these reports estimated and reported an indirect effect originating from generative AI exposure; in four as an antecedent predictor of another outcome; in three as a descriptive correlate; and in one as a moderator. This matters because a study that treats load as an outcome answers a different question from one that treats it as a mediator. The former asks whether GenAI changes load; the latter asks whether load is the mechanism through which GenAI changes learning.

Studies that positioned cognitive load as a mediator or pathway variable are nevertheless informative about proposed mechanisms. (Zhang and Jiang 2026) positioned the three load components as pathways linking AI dependency to higher-order thinking and enjoyment. That such mechanistic designs remain a small minority reinforces the review's broader point: the corpus is rich in demonstrations that GenAI moves load, but comparatively poor in tests of whether moving load is how GenAI affects learning.

### Learning outcomes co-measured with load

Cognitive load was rarely studied in isolation; almost every study co-measured a learning outcome, and the choice of outcome shapes what a load finding means. Across the corpus, the most frequent co-measured outcomes were academic performance or achievement, higher-order thinking, self-efficacy and motivation, engagement, and self-regulation or metacognition. This co-measurement is analytically important because the worrying scenario—GenAI lowering load by displacing cognition—predicts a specific dissociation: load falls while a deep-learning outcome such as higher-order thinking fails to rise or declines.

Several studies illustrate how outcome choice disambiguates a load result. When reduced extraneous load accompanied improved performance and higher germane load—as in Jing et al. (2025) under self-paced AIGC-supported questioning and Sun and Liu (2026b)—the case for genuine learning was stronger. Joo et al. (2026) found no significant group effect on cognitive load and no retention-test difference, although the prompted group showed more distributed problem-solving activity and outperformed the comparison group on the second problem-solving task. Xiao et al. (2025) likewise reported that AI-enhanced characters ‘did not increase students' cognitive load,' but the intervention also improved writing performance. These mixed profiles show why a null load effect should not be treated as a null learning effect. Load findings acquire pedagogical meaning only in conjunction with the outcomes alongside which they are measured, which further supports designs that report load and learning together.

### The net verdict: conditional rather than uniform benefit

When each study's overall stance on GenAI for cognitive load is considered, the modal verdict is conditional. Twenty-one studies concluded that GenAI's benefits for cognitive load are contingent—on scaffolding, dosage, task design, or learner prior knowledge (Zhang and Jiang, 2026; Chen et al., 2026; Li et al., 2024)—while 16 reported an essentially beneficial effect and two were mixed (Joo et al., 2026; Xiao et al., 2025). The prevalence of conditional verdicts is consistent with the extraneous-load evidence reviewed above: GenAI reliably lowers load when it reduces coordination and presentation demands, but the same technology can raise load when it adds verification burden or invite disengagement when it supplants effortful processing.

**Figure 6:**
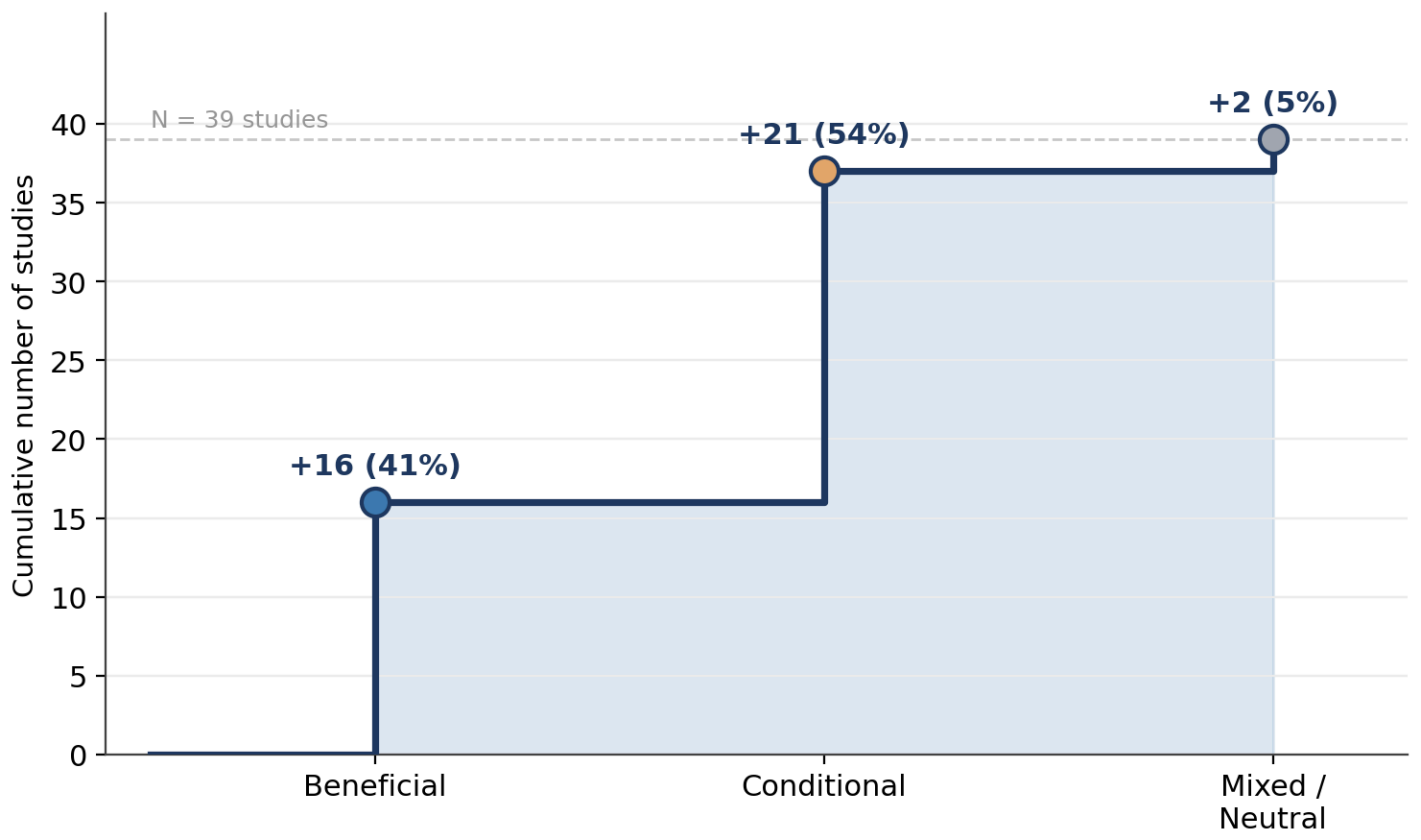
Cumulative distribution of studies' net verdicts on generative AI for cognitive load.

The conditional consensus thus reframes the practical question. The issue is not whether GenAI is good or bad for cognitive load in the abstract, but under what design conditions it relieves the right load. Structured support, worked examples, and metacognitive prompting recur across the beneficial and conditional studies as the features that tip the balance toward load relief with learning preserved (Jing et al., 2025; Sun and Liu, 2026b); unstructured, open-ended reliance recurs as the configuration that risks either added coordination cost or displaced cognition. As Figure 6 displays, conditional and beneficial verdicts together account for almost the entire corpus, but the predominance of the conditional category is the more important signal.

## Discussion

Synthesized through the CLT spine, the 39 studies yield a picture that is promising but unresolved. Within the 16 studies that measured extraneous load as a distinct subtype, lower extraneous load was reported more often than higher load. The separate overall-load evidence points in a similar descriptive direction, but it cannot tell whether the reduction reflects lower extraneous demands or reduced deep processing. The review's contribution is therefore diagnostic: it identifies where a seemingly simple conclusion about ‘lower cognitive load' exceeds what the measures can support.

The central unresolved issue is not that germane load is wholly absent, but that directional comparative evidence is sparse. Twenty-eight studies did not measure a germane subscale, and six of the 11 studies that did include one did not provide a directional quantitative GenAI comparison. Reported reductions in overall load are consequently ambiguous between freeing capacity for learning and offloading the learning itself. The studies that adopt an offloading framing perceive this hazard directly (Liu D. et al., 2026; Xu X. et al., 2025), but they remain a minority. The existing corpus does not yet permit a field-wide answer to whether GenAI preserves or displaces the effortful cognition associated with learning.

These measurement and framing gaps take on added significance in light of recent scholarship arguing that AI may redistribute, rather than simply reduce, the cognitive labor of educational work. Rind (2026), applying the same tripartite CLT lens to teacher expertise, argues that AI can offload the intrinsic and germane processing through which pedagogical expertise is built, raising the prospect of pedagogical deskilling through disuse; Rind and Qureshi (2026) report empirically that AI-mediated teaching can improve efficiency while weakening diagnostic reasoning, instructional sequencing, and reflective judgement. Read alongside our finding that germane load is almost never measured, this work sharpens the review's central concern: a technology that lowers load may be hollowing out the effortful cognition on which durable learning and expertise depend, and current measurement practice cannot detect the difference.

This argument extends rather than contradicts the instructional-design tradition within CLT. That tradition has always held that the goal is not to minimize load but to reallocate it—to suppress extraneous load so that germane processing can proceed. GenAI complicates this prescription because it can suppress extraneous and germane load simultaneously, and an overall load measure cannot tell the difference. The curvilinear dependency finding sharpens the point: maximal reliance on GenAI minimized neither extraneous load nor higher-order outcomes, with the optimum at moderate use (Zhang and Jiang, 2026). Such non-monotonicity is exactly what an offloading account predicts, since beyond some threshold the learner ceases to do the cognitive work that load was meant to index.

The recurrent conditional verdicts should be treated as a research agenda rather than as a pooled causal conclusion. Across studies, scaffolding, metacognitive prompting, learner prior knowledge, and dosage recur as plausible moderators, but their effects are not measured in a sufficiently comparable way for precision-weighted synthesis. The practical implication is that the configuration of GenAI support matters more than the presence of GenAI alone; future studies need to test these design conditions while separately measuring the CLT components.

The findings also speak to a tension between subjective and objective indices of cognitive cost. Most studies in the corpus relied on self-reported load, and self-report is precisely the channel through which offloading is most likely to mislead: a learner who has delegated the hard thinking to GenAI will, accurately, report that the task felt easy. Several studies hint at the value of converging measures—Xu T. et al. (2025) used eye-tracking alongside self-report, and the survey and experimental traditions could be bridged by pairing load scales with performance and process data. The lesson is not that self-report is invalid but that, for a technology capable of substituting for cognition, a low subjective load is a necessary but not sufficient sign of good instructional design. Without an accompanying germane-load measure or a transfer test, ease and learning cannot be told apart.

Methodologically, the review exposes a comparability problem. The dispersion of instruments, the prevalence of undifferentiated load indices, inconsistent causal roles assigned to load, and absence of a formal study-level risk-of-bias appraisal mean that ostensibly similar findings cannot be pooled or weighted with confidence. Future synthesis—and especially any future meta-analysis—will require separate reporting of the CLT components, transparent intervention and comparison conditions, study-level appraisal, and effect estimates suitable for synthesis.

## Limitations

Several limitations bound the interpretation of this review. First, the search was restricted to English-language articles indexed in the SSCI within the Web of Science Core Collection and the Education and Educational Research category; studies indexed elsewhere may therefore be absent, and the conclusions apply to this defined corpus rather than the entire field. Second, the heterogeneity of instruments and study designs, together with the absence of a validated independent study-level risk-of-bias appraisal, precludes quality- or precision-weighted synthesis. Third, most studies relied on self-report and many used overall or undifferentiated load measures, so lower reported load cannot by itself be interpreted as lower extraneous load or improved learning.

## Implications

For practitioners, the review's clearest implication is that the design of GenAI support, not its mere adoption, determines its cognitive consequences. Structured uses—worked examples, decomposition, and metacognitive prompting—are associated with relieved extraneous load and preserved learning, whereas open-ended reliance risks either added coordination cost or displaced cognition. Instructors should therefore scaffold how learners engage GenAI and should be wary of treating a subjective sense of ease as evidence of learning. For researchers, the implications are methodological and theoretical: studies should measure the intrinsic, extraneous, and germane components separately rather than reporting an overall index, should adopt the cognitive-offloading lens to interpret load reductions, and should pair self-report with process or performance measures capable of detecting whether deep processing has occurred. Doing so would let the field distinguish GenAI that makes room for learning from GenAI that substitutes for it—a distinction the current evidence base is structurally unable to draw.

Whether a learner offloads productively or dependently is, moreover, not only a matter of task design but of the learner's epistemic stance toward the tool. Rind et al. (2026) trace how students move from blind trust toward critical inquiry when engaging ChatGPT in higher education, implying that epistemic beliefs, calibrated trust, and the disposition to verify AI output moderate whether a load reduction reflects support or substitution. Future studies of GenAI and cognitive load should therefore incorporate metacognitive scaffolding, verification practices, and claim-evidence reasoning as measured moderators rather than as design features alone. Teacher preparation, digital competence, and contextual constraints also shape educational technology use more broadly (Soomro et al., 2025).

## Conclusion

This systematic review mapped 39 empirical WoS/SSCI-indexed studies of generative AI and closely related educational AI systems through the tripartite architecture of Cognitive Load Theory, extended with the cognitive-offloading perspective. Three conclusions follow. First, lower reported load is more common than higher reported load in the distinct-extraneous and overall/undifferentiated measurement groups, but the groups cannot be combined as evidence that AI or GenAI reduces extraneous load specifically. Second, only 11 studies included a germane-load subscale and only five reported a directional comparative result, leaving the distinction between productive load reallocation and displaced learning largely unresolved. Third, the cognitive-offloading lens was adopted in only 13 studies, leaving the mechanism of reported load reduction under-theorized.

The modal verdict of the corpus—conditional benefit, coded in 21 of 39 studies—captures the present state of knowledge: the cognitive consequences of GenAI depend on task design, scaffolding, learner knowledge, and use conditions. This review should not be read as a precision-weighted estimate of GenAI's effect, because the evidence base uses heterogeneous measures and lacks a preserved formal study-level appraisal. The next generation of research should report the CLT triad separately, measure germane load and deep-learning outcomes, specify the GenAI comparison and verification demands, and pair subjective load with process or performance evidence. Only then can the field answer the central question: not merely whether GenAI makes a task feel easier, but whether it frees capacity for learning rather than performing the learning in the learner's place.

## Supplementary material

Supplementary Tables S1–S3 Included studies, full-text exclusions, and study-level design and measurement characteristics.

Supplementary File PRISMA 2020 checklist.
