# Supplementary material for: Generative AI and cognitive load in education: a systematic review of WoS/SSCI-indexed studies through the lens of cognitive load theory
Source: Front Psychol. 2026 Aug 27;17:1921504. doi: 10.3389/fpsyg.2026.1921504 (PMC13560642; doi:10.3389/fpsyg.2026.1921504)
Supplement: Supplementary file 1 [file Supplementary_file_2.docx]

# Supplementary File S4. PRISMA 2020 Checklist

Manuscript: Generative AI and Cognitive Load in Education: A Systematic Review of WoS/SSCI-Indexed Studies Through the Lens of Cognitive Load Theory. This checklist identifies where each PRISMA 2020 reporting item is addressed. Items that were not applicable or methods that were not performed are stated explicitly.

| **Item** | **Topic** | **Checklist item** | **Location in this manuscript** |
| --- | --- | --- | --- |
| 1 | Title | Identify the report as a systematic review. | Title |
| 2 | Abstract | PRISMA 2020 for Abstracts. | Abstract |
| 3 | Rationale | Describe the rationale for the review. | Introduction |
| 4 | Objectives | Explicit objectives/questions. | Introduction: RQ1-RQ3 |
| 5 | Eligibility criteria | Inclusion and exclusion criteria. | Method: Inclusion criteria |
| 6 | Information sources | Databases and date of last search. | Methods - Search strategy: Web of Science Core Collection; final search reported as June 2026; index, language, document-type, category, and publication-year coverage stated. |
| 7 | Search strategy | Full search strategies. | Methods - Search strategy: the three Topic-field entries, AND logic, filters, and saved Web of Science access link are reported; the saved link is not the sole audit record. |
| 8 | Selection process | Methods to decide inclusion. | Methods - Selection and coding; Figure 1; Supplementary Table S2 lists all 19 database-path full-text exclusions and item-level reasons. |
| 9 | Data collection process | Methods to collect data from reports. | Method: coding; dual coding |
| 10a | Data items | Outcomes defined. | Method: 14 dimensions; Results |
| 10b | Data items | Other variables defined. | Method: design, sample, instrument, roles, verdict |
| 11 | Study risk of bias assessment | Quality / risk-of-bias methods. | No validated formal study-level risk-of-bias or methodological-quality tool was applied. This is reported in the Methods and Limitations; Supplementary Table S3 provides descriptive design and measurement characteristics only. |
| 12 | Effect measures | Effect measure(s). | No common effect measure was specified because the review presents a structured descriptive evidence map and does not pool effect estimates. |
| 13a | Synthesis methods | Eligibility for each synthesis. | Method: Synthesis |
| 13b | Synthesis methods | Data preparation. | Method: forced-choice coding; consensus |
| 13c | Synthesis methods | Tabulation/display. | Results; Figures 2-6; Table S1 |
| 13d | Synthesis methods | Statistical synthesis/heterogeneity. | No statistical meta-analysis was conducted. Instrument, intervention, comparison, and effect-metric heterogeneity, and the descriptive synthesis approach, are reported in the Methods and Limitations. |
| 13e | Synthesis methods | Sensitivity analyses. | No formal sensitivity analysis was conducted. Separating measurement groups is a reporting correction, not a quality-based sensitivity analysis. |
| 13f | Synthesis methods | Certainty methods. | No certainty-of-evidence method was applied. |
| 14 | Reporting bias assessment | Missing-results bias methods. | No formal missing-results or reporting-bias assessment was conducted; the single-source coverage limitation is discussed. |
| 15 | Certainty assessment | Certainty in body of evidence. | No formal certainty-of-evidence assessment was conducted. |
| 16a | Study selection results | Search and selection results. | Methods and Figure 1: 139 records identified; 53 removed before screening; 86 screened; 30 excluded at title/abstract; 56 assessed in full text; 19 excluded; 37 database-path studies plus 2 backward-citation studies included (N = 39). |
| 16b | Study selection results | Excluded studies with reasons. | Supplementary Table S2 lists all 19 database-path full-text exclusions and gives an item-level eligibility reason for each report. |
| 17 | Study characteristics | Cite included studies and characteristics. | Supplementary Tables S1 and S3 provide full citations and study-level design and measurement characteristics for all 39 included studies. |
| 18 | Risk of bias in studies | Present assessments. | No formal study-level risk-of-bias assessment was conducted. Supplementary Table S3 provides descriptive design and measurement characteristics only. |
| 19 | Results of individual studies | Per-study summary stats/effects. | Supplementary Table S1 provides study-level design, measurement, direction, and verdict information. Comparable per-study effect estimates were not available for a uniform display. |
| 20a | Results of syntheses | Summarize synthesis results. | Results (RQ2-RQ3): 21 conditional, 16 beneficial, and 2 mixed overall verdicts; measurement-group denominators are reported separately. |
| 20b | Results of syntheses | Heterogeneity. | Results/Discussion |
| 20c | Results of syntheses | Sensitivity results. | No formal sensitivity analysis was conducted; see item 13e. |
| 20d | Results of syntheses | Certainty results. | No formal certainty-of-evidence assessment was conducted. |
| 21 | Reporting biases | Present reporting-bias assessments. | No formal reporting-bias assessment was conducted. Single-source and archival limitations are discussed. |
| 22 | Certainty of evidence | Present certainty assessments. | No formal certainty-of-evidence assessment was conducted. |
| 23a | Discussion | General interpretation. | Discussion |
| 23b | Discussion | Limitations. | Limitations |
| 23c | Discussion | Implications. | Implications; Conclusion |
| 24a | Registration/protocol | Registration information. | Methods: the review was not preregistered. |
| 24b | Registration/protocol | Protocol access. | Not applicable: the review was not preregistered and no protocol is available. |
| 24c | Registration/protocol | Protocol amendments. | Not applicable: no protocol amendments were applicable because no protocol was registered. |
| 25 | Support | Sources of support. | See manuscript funding statement |
| 26 | Competing interests | Declare competing interests. | See manuscript COI statement |
| 27 | Availability of data | Data/code/materials availability. | Supplementary Tables S1-S3 and the supplementary Search record are provided with the submission. The saved Web of Science query URL is an access aid and is not the sole audit record. |

*Note. Checklist adapted from the PRISMA 2020 statement (Page et al., 2021). Items that were not applicable or methods that were not performed are identified explicitly.*
